# Anion Exchange Membranes Based on Imidazoline Quaternized Polystyrene Copolymers for Fuel Cell Applications

**DOI:** 10.3390/membranes11110901

**Published:** 2021-11-22

**Authors:** Li-Cheng Jheng, Chung-Yen Hsu, Hong-Yi Yeh

**Affiliations:** Department of Chemical and Materials Engineering, National Kaohsiung University of Science and Technology, Kaohsiung 80778, Taiwan; j841216@gmail.com (C.-Y.H.); F109146121@nkust.edu.tw (H.-Y.Y.)

**Keywords:** imidazoline, polystyrene, anion exchange membranes, fuel cells

## Abstract

Imidazoline is a five-membered heterocycle derived by the partial reduction of one double bond of the imidazole ring. This work prepared new anion exchange membranes (AEMs) based on imidazoline quaternized polystyrene copolymers bearing N-b-hydroxyethyl oleyl imidazolinium pendent groups to evaluate the application potential for anion exchange membrane fuel cells (AEMFCs). For comparison, an imidazole quaternized polystyrene copolymer was also synthesized. The polymer chemical structure was confirmed by FTIR, NMR, and TGA. In addition, the essential properties of membranes, including ion exchange capacity (IEC), water uptake, and hydroxide conductivity, were measured. The alkaline stabilities of imidazolium-based and imidazolinium-based AEMs were compared by means of the changes in the TGA thermograms, FTIR spectra, and hydroxide conductivity during the alkaline treatment in 1 M KOH at 60 °C for 144 h. The results showed that the imidazolinium-based AEMs exhibited relatively lower hydroxide conductivity (5.77 mS/cm at 70 °C) but much better alkaline stability compared with the imidazolium-based AEM. The imidazolinium-based AEM (PSVBImn-50) retained 92% of its hydroxide conductivity after the alkaline treatment. Besides, the fuel cell performance of the imidazolium-based and imidazolinium-based AEMs was examined by single-cell tests.

## 1. Introduction

Proton exchange membrane fuel cell (PEMFC) is the main type of fuel cell that can provide clean energy for many portable and transportation applications. However, the high cost of PEMFC resulting from the use of noble metal-based catalysts still is a major obstacle for its commercialization [1,2]. Alternative to PEMFC, an anion exchange membrane fuel cell (AEMFC) having faster oxygen reduction kinetics allows platinum-free catalysts (e.g., Ag, Ni, Co, and Fe). Meanwhile, the use of metal-based bipolar plates and lower fuel permeability are other advantages of AEMFC over PEMFC [3,4,5].

Anion exchange membrane (AEM) is one of the main components of AEMFC, which is capable of conducting anions and avoiding gas penetration from one electrode side to another side. Typically, AEMs are constituted by polymers containing cationic groups. However, there is a challenge for an AEM to maintain the ion exchange capacity (IEC) and ionic conductivity in the presence of hydroxide ions because most cationic groups (e.g., quaternary ammonium, imidazolium, and benzimidazolium) are susceptible to degrade at an elevated temperature (>60 °C) under strongly basic conditions [6,7].

The alkaline stability of AEMs could be influenced by a variety of factors, including cation structure, polymer backbone structure, steric hindrance, and crosslinking [3]. Among them, the influence of cation structure usually is more decisive [8]. Many efforts have been made to covalently tether novel cations with high alkaline stability on the polymer backbone of the AEM [9,10,11,12,13]. Some alkaline stable cations such as guanidinium [14], piperidinium [15], quinuclidinium [16], pyrrolidinium [17], and morpholinium [18] have been suggested to be used in AEMs for fuel cell applications. In addition to cation structure, the polymer backbones play an important role in chemical stability. For example, aryl ether-containing polymer backbones, such as polyarylene ether sulfone (PAES), polyether ether ketone (PEEK), and polyphenyl oxide (PPO), may undergo degradation in alkaline media [19]. Furthermore, previous studies suggested that polymer backbone structure can also bring a direct influence on the alkaline stability of cationic groups [3,20]. Unlike PAES and PPO, polystyrene would not make the pendent cationic groups less stable against hydroxide attack, as Hickner and his co-worker reported [20].

**Scheme 1:**
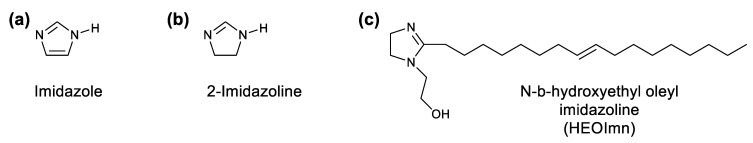
Chemical structures of (**a**) imidazole, (**b**) 2-imidazoline, and (**c**) N-b-hydroxyethyl oleyl imidazoline (HEOImn).

Imidazoline is a nonplanar and five-membered heterocycle. The chemical structure difference between imidazole and 2-imidazoline (4,5-dihydroimidazole) is whether the C=C doubt bond of heterocycle is reduced or not, as shown in Scheme 1. Imidazoline compounds with an alkyl chain on the C2 position are the most common type used in the industry, such as the commercially available N-b-hydroxyethyl oleyl imidazoline (HEOImn). The cationic imidazoline compounds (i.e., imidazolinium) can be used in various industrial applications as corrosion inhibitors, emulsifiers, dispersing agents, adhesion promoters, lubricants, and textile softeners [21,22,23]. Unlike how imidazoline is prone to hydrolyze in alkaline aqueous solution [24], imidazolinium seems to be relatively unaffected by pH changes [21]. This advantage makes imidazolinium-based salts useful for industrial applications.

In recent years, imidazolinium-based ionic liquids exhibiting adequate ionic conductivities have been confirmed [25,26]. However, the application potential of imidazolinium-based AEMs used in fuel cells still needs to be demonstrated. This work synthesized imidazoline quaternized polystyrene copolymers by reacting styrene-co-vinylbenzyl chloride copolymers (PSVBC) with HEOImn via the Menshutkin reaction. For comparison, an imidazole quaternized polystyrene copolymer synthesized from 1-methylimidazole and PSVBC was also prepared. The chemical structure of as-prepared polymers was characterized by Fourier transform infrared (FTIR), nuclear magnetic resonance (NMR), and thermogravimetric analysis (TGA) techniques. In addition, the essential properties of AEMs, including ion exchange capacity (IEC), water uptake, and hydroxide conductivity, were investigated. The alkaline stabilities of imidazolium-based and imidazolinium-based AEMs were evaluated and compared according to the changes in the TGA thermograms, FTIR spectra, and hydroxide conductivity during the alkaline treatment in 1 M KOH at 60 °C for 144 h. Furthermore, single-cell tests were carried out to exam their AEMFC performance.

## 2. Materials and Methods

### 2.1. Materials

Styrene (99%, Alfa Aesar) and 4-vinylbenzyl chloride (90%, Aldrich) monomers were purified before use by passing them through basic aluminum oxide (98%, Acros Organics) to remove stabilizing agents. The chemical reagents including azobisisobutyronitrile (AIBN) (99%, Otsuka chemical), 1-methylimidazole (99%, Aldrich), N-b-hydroxyethyl oleyl imidazoline (HEOImn) (99%, Chem Service), and potassium hydroxide (>85%, Acros Organics) were used without further purifications. All the solvents used in this work such as toluene (Sigma Aldrich), dimethyl sulfoxide (DMSO) (Acros Organics), anhydrous methanol (Macron Fine Chemicals), isopropyl alcohol (Macron Fine Chemicals), chloroform (Sigma Aldrich) were ACS reagent grade. The standard solutions for the titration analysis, 0.1 M sodium hydroxide solution and 0.1 M hydrochloric acid solution, were purchased from Honeywell Fluka. Nitrogen, hydrogen, and oxygen gases with a high purity of greater than 99.99% were provided from local manufacturer (Jing-Shang gas).

### 2.2. Syntheses of Quaternized Polystyrene Copolymers

The synthesis procedures for preparing two polystyrene copolymers in different feed ratios were described below according to a previous method [7]. A predetermined amount of styrene (70 or 50 mmol), vinylbenzyl chloride (VBC) (30 or 50 mmol), AIBN (0.5 mmol), and toluene (15 mL) was placed in a three-necked flask equipped with a condenser. The mixture was heated to 70 °C from room temperature with an oil bath. During the polymerization, the mixture was stirred for 16 h under a nitrogen atmosphere at 70 °C. After that, the mixture was poured into an excess amount of methanol (about 150 mL) under stirring. Subsequently, the copolymer was precipitated from the solution. After isolating from the solution, the resulting copolymer was dried under a vacuum at 70 °C for 8 h to remove residual solvent. Finally, polystyrene copolymers in the styrene/VBC molar ratios of 7:3 and 5:5 can be obtained. They are designated as PSVBC-30 and PSVBC-50, respectively.

The synthesis procedure for preparing imidazoline quaternized polystyrene copolymer, called PSVBImn, is described as follows. A predetermined amount of either PSVBC-30 or PSVBC-50 (1 mmol) was mixed with HEOImn in a stoichiometric ratio in DMF (30 mL) under magnetically stirring. The reaction underwent under a nitrogen atmosphere and vigorously stirring at 120 °C for 48 h. The imidazoline molecules were gradually converted into imidazolinium cations by grafting them onto polystyrene copolymer as side-groups during the reaction. After the polymer solution was cooled to room temperature, the resulting polymer was precipitated from methanol. PSVBImn powder can be obtained after drying under a vacuum at 70 °C for 12 h.

The synthesis of imidazole quaternized polystyrene copolymers, named PSVBIm, following the synthetic route similar to that of the PSBVImn copolymers. HEOImn was replaced by 1-methylimidazole, and the reaction temperature was decreased to be 100 °C.

### 2.3. Preparation of Anion Exchange Membranes Based on Quaternized Polystyrene Copolymers

The polymer solutions of quaternized polystyrene copolymers (PSBVImn and PSBVIm) obtained after the polymerizations were directly used for the membrane casting. The solid content of the polymer solution was adjusted to be approximately 5 wt% by evaporating the solvent with a rotary evaporator. The polymer solution was filtered through a 0.5 μm PTFE filter and poured onto a Teflon plate. Then, it was dried in a vacuum oven at 60 °C for 12 h under a reduced pressure to slowly evaporate most of the solvent and at 120 °C for another 12 h under vacuum to remove the rest of the solvent. After that, a polymer membrane was formed and subsequently peeled off from the plate. The membrane was washed with methanol to remove the unreacted compounds and the unremoved solvent. After drying at 60 °C for 1 h, the membrane was immersed in a 1 M KOH aqueous solution at room temperature for 24 h to convert it from the Cl^‒^ form into the OH^‒^ form. And then, it was neutralized by washing with deionized water. The membrane was stored in N_2_ saturated deionized water in a sealed vial to avoid CO_2_ contamination before use.

### 2.4. Characterization

FT-IR spectroscopy was performed on a PerkinElmer Spectrome ONE spectrometer to identify functional groups within polymer membranes. The FT-IR spectra were recorded by scanning 16 times in a transmittance mode and a wavelength range between 400 cm^−1^ and 2000 cm^−1^ with a resolution of 2 cm^−1^. NMR spectroscopy was conducted on a Bruker AVANCE 600 MHz spectrometer to confirm the chemical structure of polymers synthesized in this work. CPC traces were recorded on a GPC system consisting of a Hitachi L-2490 IR Detector and a Hitachi L-2130 Pump to determine the molecular weight of a polymer. Before analysis, a 2 wt% polymer solution in THF was prepared and filtered through a 0.45 m PTFE filter. The GPC analysis was carried out using THF as the eluent at an elution rate of 0.5 mL/min and polystyrene as the calibration standards. TGA was used to examine the thermal degradation of a polymer with increasing temperature. The thermograms were recorded on a TA SDT-Q600 instrument at a heating rate of 10 °C/min from 100 °C to 700 °C with a 20 mL/min nitrogen flow.

### 2.5. Measurements

The ion exchange capacity (IEC) of an anion exchange membrane was measured by the back titration method. Before measurement, the OH^‒^ form membrane was dried in a vacuum oven at 100 °C for 3 h. After weighing the dried membrane, it was immersed in a 0.1 M HCl standard solution for 24 h at room temperature to replace all OH^‒^ ions in the membrane with Cl^‒^ ions. Afterward, the membrane was back-titrated with a 0.1 M NaOH standard solution using phenolphthalein as an indicator. The experimental IEC is determined using the following equation:

(1) $$\mathrm{IEC}=\frac{V_{o,\mathrm{NaOH}}\times C_{\mathrm{NaOH}}-V_{\mathrm{NaOH}}\times C_{\mathrm{NaOH}}}{m_{\mathrm{dry}}}$$

where V_o,NaOH_ and V_NaOH_ are the volume of the NaOH solution consumed in the titration without and with a membrane, respectively, C_NaOH_ is the molar concentration of the NaOH standard solution, m_dry_ is the mass of the dried membrane in OH^‒^ form.

The weight and length differences of a OH form^‒^ membrane between in the dry state and in fully hydrated state can determine its water uptake (WU) and swelling ratio (SR), respectively. The membrane sample was immersed in N_2_ saturated deionized water in a sealed vial at 60 °C for 24 h. After picking out the membrane from the vial and removing surface water, the membrane’s weight and length in the fully hydrated state (*W_h_* and *L_h_*) were measured. Then, it was dried in a vacuum oven at 100 °C for 3 h and followed by measuring its weight and length in the dry state (*W_d_* and *L_d_*). The membrane’s WU and SR can be calculated as follows:

(2) $$\mathrm{WU}=\frac{W_{h}-W_{d}}{W_{d}}$$

(3) $$\mathrm{SR}=\frac{L_{h}-L_{d}}{L_{d}}$$

The gel fraction refers to the content of the insoluble part in an anion exchange membrane, which was evaluated using the Soxhlet method in this work. At first, the membrane was weighed in a dry state after drying under vacuum at 100 °C for 3 h. The membrane sample was subsequently placed in a Soxhlet apparatus and refluxed with DMF at the boiling temperature for 24 h. After washing with methanol and drying under vacuum at 50 °C for 12 h, the membrane sample was weighed again. The gel fraction can be obtained by comparing the membrane’s weight change before and after the Soxhlet extraction as the following relation.

(4) $$\mathrm{Gel}\;\mathrm{fraciotn}=\frac{W_{d}-W_{d,\;\;extracted}}{W_{d}}$$

The terms *W_d_* and *W_d,extraction_* are referred to as the membrane’s weight in the dry state before and after the extraction, respectively.

The ionic conductivity (σ) of an anion exchange membrane was measured using a 4-electrode impedance method. The membrane sample was cut into a dimension of 30 × 4.5 mm (length × width) and was inserted into the 4-electrode cell (BekkTech BT-112) immersed in N_2_ saturated deionized water in a sealed flask. The cell was connected to an Autolab PGSTAT128N impedance analyzer to perform the ionic conductivity measurement in a frequency range between 10^2^ Hz and 10^5^ Hz with an alternating voltage amplitude of 10 mV. The measured temperature of ionic conductivity can be adjusted by controlling the water temperature. The ionic conductivity was determined using the following equation.

(5) $$\sigma =\frac{L}{A\times R}$$

where σ, *L*, *A*, and *R* are the ionic conductivity of the membrane in the unit of S cm^−1^, the length between the two voltage measuring probes, the cross-sectional area perpendicular to the current flow, and the ohmic resistance of the membrane, respectively. *R* is the real impedance-axis intercept value obtained from the Nyquist plot.

For each water uptake and swelling ratio value, the measurement was repeated 5 times. In addition, we conducted the measurements three times to obtain the average values of experimental IEC and ionic conductivity.

### 2.6. Alkaline Stability

During alkaline treatment, the OH^‒^ form membrane sample was immersed in 1 M KOH solution at 60 °C for 144 h. The ionic conductivity at 60 °C as a function of treatment time was recorded to evaluate the membrane’s alkaline stability. Before each ionic conductivity measurement, the membrane sample was washed with deionized water several times to remove extra hydroxide ions on the membrane surface. In addition, the membrane’s TGA thermograms and evolution of FTIR spectra during the alkaline treatment were compared to detect alkaline degradations by identifying the changes in chemical structure or composition.

### 2.7. Single-Cell Tests

The MEA for examining the fuel cell performance was prepared by the gas diffusion electrode (GDE) method as the following procedures. A Pt/Ccatalyst (VULCAN^®^ XC-72R/Cabot, 40 wt% Pt) and an ionomer solution (Sustainion^®^ XB-7/Dioxide Materials, alkaline ionomer 5% in ethanol) were dispersed in an aqueous isopropyl alcohol solution by a bath-type ultrasonication for 10 min at room temperature to make a catalyst ink. The solid content of ionomer in the catalyst ink was 20 wt%. Before the GDE fabrication, the catalyst ink was further treated with a probe-type ultrasonication of 60W output for 5 min. Subsequently, the catalyst ink was coated onto a carbon paper (N1S1007/QuinTech) on a hot plate by drop-casting to obtain a GDE with a Pt loading of 0.8 mg cm^−1^. The GDEs used on the anode and cathode sides in this work were identical. The anion exchange membrane was sandwiched between two GDEs without hot-pressing to form the MEA before the fuel cell test. During the AEMFC operation, a fuel cell with an effective area of 4 cm^2^ was supplied with humidified hydrogen and oxygen at constant flow rates of 30 mL min^−1^ and 60 mL min^−1^, respectively. Before the performance evaluation, the fuel cell underwent the activation process at a constant cell voltage of 0.6 V for a certain period of time until the current density reached a steady state. The polarization curve of the MEA at 60 °C without any back pressure was obtained using a fuel cell test system (Tension Energy Inc., Taiwan) equipped with an electronic load unit controller.

## 3. Results and Discussion

### 3.1. Characterization of Quaternized Polystyrene Copolymers

PSVBC-30 and PSVBC-50, respectively, denote the polystyrene copolymers with the VBC feed contents of 30 and 50 mol%, which were synthesized by thermally initiated free radical polymerization. The information of their compositions and molecular weights is summarized in Table 1. The VBC contents determined from ^1^H NMR analysis were 33.4 mol% and 49.3% for PSVBC-30 and PSVBC-50, respectively. These values were close to the feed content values. The GPC result presented that they had similar number-average molecular weights. Meanwhile, their weight-average molecular weights are sufficient (>5 × 10^4^ g/mol), allowing them to fabricate the free-standing membranes. S. Vengatesan reported that polystyrene copolymers synthesized from VBC and styrene exhibit a higher polydispersity index if the VBC content is higher due to the chain transfer through VBC [27]. This can explain why the polydispersity index of PSVBC-50 was higher than that of PSVBC-30 in this work.

**Scheme 2:**
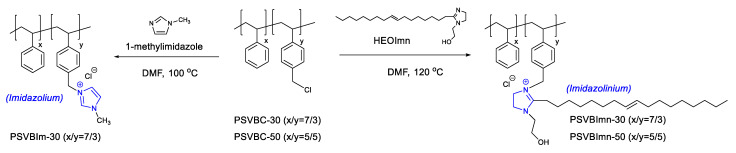
Synthetic routes of the imidazole quaternized copolymer PSVBIm-30 as well as the imidazoline quaternized copolymers PSVBImn-30 and PSVBImn-50.

Two imidazoline quaternized copolymers, referred to as PSVBImn-30 and PSVBImn-50, were synthesized by the quaternization of PSVBC-30 and PSVBC-50 with HEOImn via the Menshutkin reaction, respectively. Meanwhile, the imidazole quaternized copolymer PSVBIm-30 was prepared by reacting PSVBC-30 with 1-methylimidzole. Their synthetic routes are illustrated in Scheme 2.

**Figure 1:**
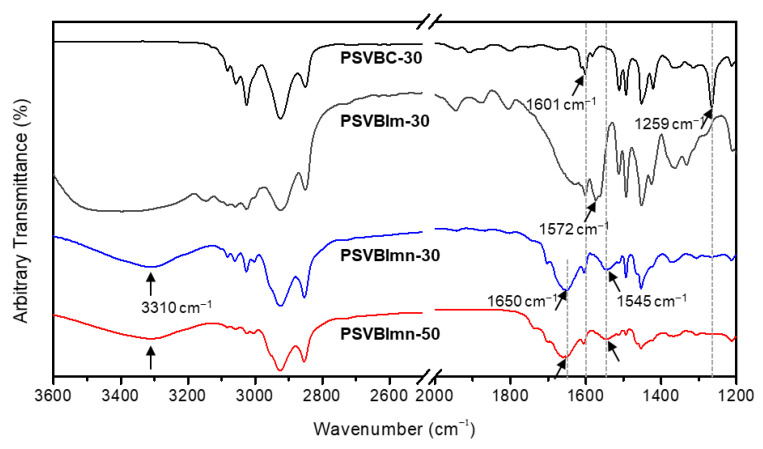
FTIR spectra of PSVBC-30, PSVBIm-30, PSVBImn-30, and PSVBImn-50.

The FTIR spectra of PSVBC-30, PSVBIm-30, PSVBImn-30, and PSVBImn-50 were presented in Figure 1. It can be observed that all the FTIR spectra displayed a band at 1601 cm^−1^ corresponding to the C=C aromatic stretching vibration as well as two bands at 2925 cm^−1^ and 2852 cm^−1^ coming from the symmetrical and asymmetrical stretching vibration of the methylene [7]. The characteristic band at 1259 cm^−1^ assigned to the CH_2_Cl wagging was only present in the spectra of PSVBC-30 but absent in the other spectra [27]. The peak at 1572 cm^−1^ corresponding to N=C-N on imidazolium appeared in the spectrum of PSVBIm-30 [7]. Meanwhile, two bands at 1650 cm^−1^ and 1545 cm^−1^ were found in both spectra of PSVBImn-30 and PSVBImn-50, which were attributed to C=N and N=C-N stretching vibrations for imidazolinium, respectively [22,28]. These findings suggested that the PSVBC copolymers can be quaternized with the 1-methylimidazole and the imidazoline compound HEOImn. In addition, we observed a two-bends band at around 3310 cm^−1^, which would be associated with the OH group for PSVBImn-30 and PSVBImn-50.

**Figure 2:**
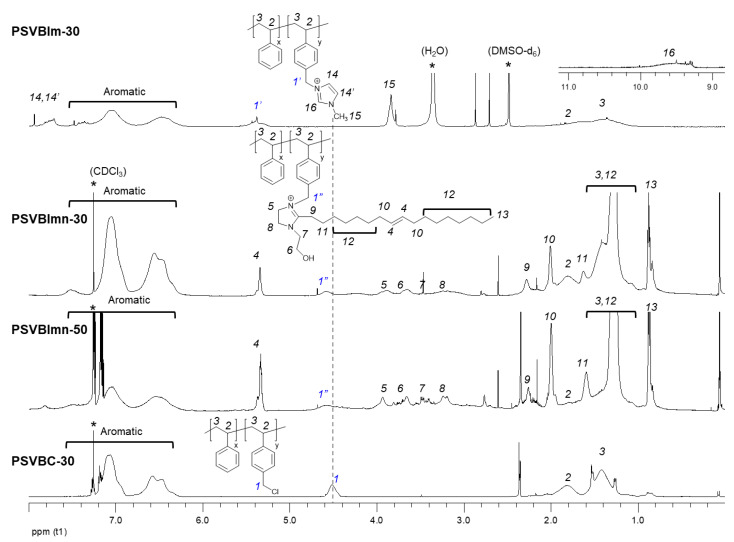
^1^H NMR spectra of PSVBC-30, PSVBIm-30, PSVBImn-30, and PSVBImn-50.

The comparative analysis of ^1^H NMR spectra for the synthesized copolymers is shown in Figure 2. The characteristic signal at the chemical shift of 4.52 ppm (H_1_) was attributed to the aliphatic methylene protons adjacent to the chloride side group for PSVBC-30 [7]. This characteristic signal was found to relocate at 5.38 ppm (H_1′_) for PSVBIm-30. In addition, it shifted toward down-field slightly at 4.59 ppm (H_1″_) for PSVBImn-30 and PSVBImn-50. These findings revealed that the quaternization of the starting copolymer with either 1-methylimidazole or HEOImn occurred. The signal at 5.35 ppm (H_4_) corresponded to the 1-ethylene protons in the long aliphatic chain of HEOImn. The signals related to the methylene protons of HEOImn were assigned in the chemical shift range of 1.0 and 4.0 ppm (H_5_, H_6_, H_7_, H_8_, H_9_, H_10_, H_11_, H_12_) [23], as seen in Figure 2. Meanwhile, the signal coming from the methyl protons of HEOImn appeared at the chemical shift of 0.89 ppm (H_13_). The methine protons of imidazolium (H_14_, H_14′_) for PSVBIm-30 were associated with the signals ranging from 7.5 and 8.0 ppm. The methyl protons (H_15_) and the C2 protons (H_16_) on PSVBIm-30 were related to the signal at 3.84 ppm and the broad signal between 10 and 9.2 ppm. The joint results of FTIR and ^1^H NMR allow us to confirm that the quaternized polystyrene copolymers (PSVBIm-30, PSVBImn-30, and PSVBIm-50) were successfully synthesized.

### 3.2. Properties of Anion Exchange Membranes Based on Quaternized Polystyrene Copolymers

The AEMs based on PSVBIm-30, PSVBImn-30, and PSVBImn-50 copolymers were prepared by a solvent casting method followed by an ion-exchange procedure. Ion exchange capacity (IEC), determined by the density of ion-conducting sites within the membrane, is crucial for an AEM and highly related to its ion conduction performance [29]. Table 2 lists the results of IEC, water uptake, swelling ratio, and gel fraction for the OH^‒^ form AEMs prepared in the present work. As predicted, the experimental IEC of PSVBIm-30 (1.88 mmol g^−1^) was higher than those of PSVBImn-30 (1.11 mmol g^−1^) and PSVBImn-50 (1.63 mmol g^−1^). Their experimental IECs measured using a back-titration method agreed with their theoretical IEC values individually, indicating that the quaternization and the ion-exchange process of the AEMs were completed.

It is known that the IEC value influences both the water uptake (WU) and swelling ratio (SR) for an AEM in the hydrated state. Generally, the quaternized PSVBC copolymer with a VBC molar content up to 50 mol% does not have acceptable dimensional stability in fully hydrated condition, especially for its OH^‒^ form membrane. For example, S. Vengatesan et al. observed that the quaternary ammonium functionalized PSVBC in the styrene to VBC feed ratio of 1:1 (namely, 50 mol% VBC content) crumbled in water. This phenomenon happened due to the high amount of hydrophilic ionic groups on the copolymer [27]. In our case, we failed to prepare an AEM based on imidazole quaternized PSVBIm-50, which lost its dimensions immediately once immersed in water at 60 °C. However, it is surprising that imidazoline quaternized PSVBImn-50 exhibited good dimensional stability in its fully hydrated state. The SR of the PSVBImn-50 membrane was measured at a relatively low value of 13.9%, while its WU was as high as 210.7%. It was found that both PSVBImn-30 and PSVBImn-50 membranes grafted with HEOImn cannot re-dissolve in polar organic solvents again after the solvent-casting process, indicating unexpected polymer crosslinking between their unsaturated oleyl groups. We suppose that the good dimensional stability of the PSVBImn-50 membrane was associated with the formation of crosslinking networks.

Compared with the PSBVIm-50 and PSVBImn-50 membranes, the PSVBImn-30 membrane with a lower IEC exhibited a lower WU (84.6%) and a lower SR (4.0%) at 60 °C as expected. However, the WU of PSVBImn-50 was higher than that of PSVBIm-30, although PSVBImn-50 had a relatively low IEC. The hydroxyl group on HEOImn may be responsible for the better water absorption of PSVBImn-50.

**Scheme 3:**
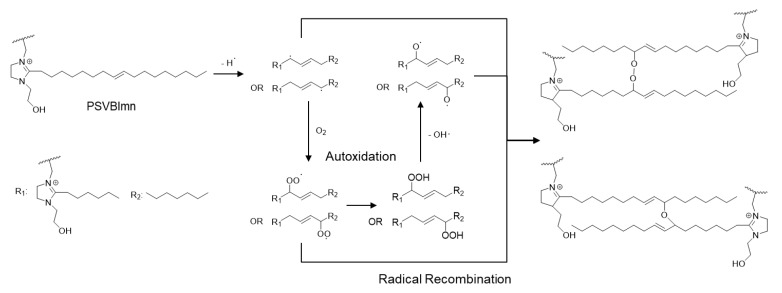
Proposed mechanisms for the crosslinking of imidazoline quaternized polystyrene polymers with unsaturated oleyl chains.

To evaluate the crosslinking level, the gel fractions of these AEMs were measured. The result showed that the gel fractions of the PSVBIm-30, PSVBImn-30 and PSVBImn-50 membranes were 0%, 30% and 55%, respectively. This result confirmed the minor crosslinking occurred within the membranes for PSVBImn-30 and PSVBImn-50, which effectively restricted the polymer chain motions and allowed the membranes to remain dimension stable at a relatively high water uptake level. The possible crosslinking mechanisms for the imidazolinium functionalized polymers with unsaturated oleyl chain during the synthesis and membrane-casting processes is illustrated in Scheme 3. Basically, the formation of crosslinked networks would consist of two major steps, involving the autoxidation of unsaturated oleyl chain and the subsequent radical recombination [30]. The actual crosslinking process may be more complicated and diverse, as described in the literature [31,32,33].

### 3.3. Ionic Conductivity of the Anion Exchange Membranes

**Figure 3:**
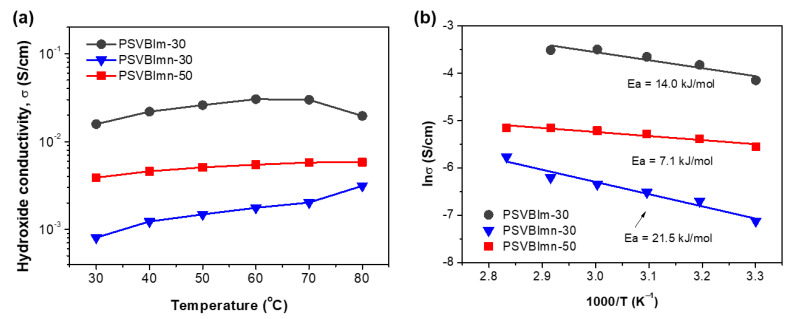
(**a**) Temperature dependence of ionic conductivity and (**b**) the Arrhenius plots for the PSVBIm-30, PSVBImn-30, and PSVBImn-50 membranes in OH^‒^ form.

The hydroxide conductivities of AEMs were measured using a four-electrode impedance method. Figure 3a shows the temperature dependence of ionic conductivity in the range from 30 °C to 80 °C for the OH^‒^ form AEMs prepared in this work. The result showed that the highest hydroxide conductivities of the PSVBIm-30, PSVBImn-30, and PSVBImn-50 membranes were measured to be 30.4 mS/cm at 60 °C, 5.8 mS/cm at 80 °C, and 3.1 mS/cm at 80 °C, respectively. It confirmed that the ionic conductivity of an AEM is greatly affected by its IEC [29]. Despite the fact that hydroxide conductivities of the PSVBImn-30 and PSVBImn-50 membranes did not exceed 10 mS/cm, this result suggested that imidazolinium cations can function as the ion-conducting sites well in polymer electrolyte membranes, such as imidazolium cations.

Conversely, it is noted that the ionic conductivity of the PSVBIm-30 membrane began to drop when the temperatures was higher than 70 °C. That may be due to the fact that PSVBIm-30 lost its dimensional stability and partially dissolved in water at relatively high temperatures.

The activation energy for hydroxide ion transport through the AEM can be obtained from the slope in the Arrhenius plot as provided in Figure 3b. The activation energies for these three AEMs were in the order of PSVBImn-50 < PSVBIm-30 < PSVBImn-30 corresponding to the ascending order of their WU values. We supposed that the more water content may allow the molecules to diffuse more efficiently in a membrane. Accordingly, the ion transport in the PSVBIm-30 and PSVBImn-50 membranes prefer via vehicular mechanism (Ea < 14 KJ/mol) instead of Grotthuss mechanism [34].

As summarized in Table 3, the hydroxide conductivity measured at 30 °C of the PSVBImn-50 membrane containing imidazolinium cations was comparable to some of those AEMs based on poly(St-co-VBC) containing quaternary ammonium or imidazolium cations, such as QMSV-0.33, Poly(St-co-VBMI), and Membrane-1 (TMA), reported in the literature [2,7,27,35]. However, the PSVBImn-50 membrane prepared in the present work could not reach a hydroxide conductivity level higher than 10 mS/cm, which would bring a negative effect on the fuel cell performance that will be described later. The commercially available imidazoline compound (HEOImn) used in this work has an oleyl chain substituted on the C2 position. The steric hindrance of both the substituted long chain and the crosslinked networks for the anion conduction, as well as the relatively low IEC, may result in the insufficient hydroxide conductivities of the PSVBImn-30 and PSVBImn-50 membranes.

### 3.4. Alkaline Stability of the Anion Exchange Membranes

**Figure 4:**
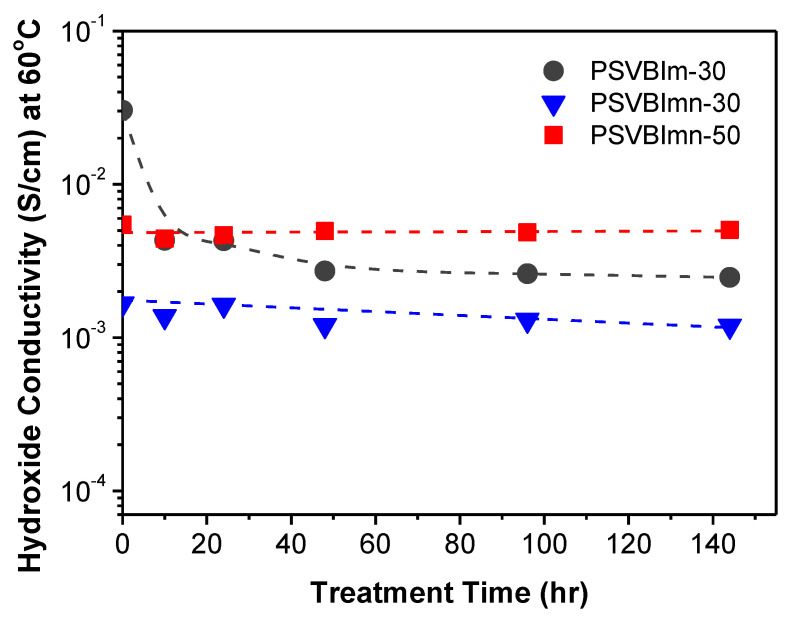
Ionic conductivities measured at 60 °C of the PSVBIm-30, PSVBImn-30, and PSVBImn-50 membranes as a function of time during the alkaline treatment in 1 M KOH at 60 °C for 144 h.

The alkaline stabilities of the AEMs based on imidazoline quaternized or imidazole quaternized polystyrene copolymers were compared and evaluated through hydroxide conductivity, TGA, and FTIR analyses after the alkaline treatment in 1 M KOH at 60 °C for 144 h. Figure 4 shows the change in hydroxide conductivity at 60 °C of the PSVBIm-30, PSVBImn-30, and PSVBImn-50 membranes as a function of time. The loss in hydroxide conductivity during the alkaline treatment was substantial for PSVBIm-30 but relatively slight for both the PSVBImn-30 and PSVBImn-50 membranes. The hydroxide conductivity of the PSVBIm-30 membrane was decreased by 91.1% to 2.5 mS/cm, which was even lower than that of the PSVBImn-50 membrane (5.05 mS/cm) after 144 h. It is noted that the PSVBImn-30 membrane lost 28.4% of its hydroxide conductivity, and the PSVBImn-50 membrane remained 92% of the initial value. This result revealed that the imidazolinium-based AEMs avoided the alkaline degradations better than the imidazolium-based AEMs did. More specifically, N-b-hydroxyethyl oleyl imidazolinium cations are supposed to be more alkaline stable than 1-methylimidazolium cations.

**Figure 5:**
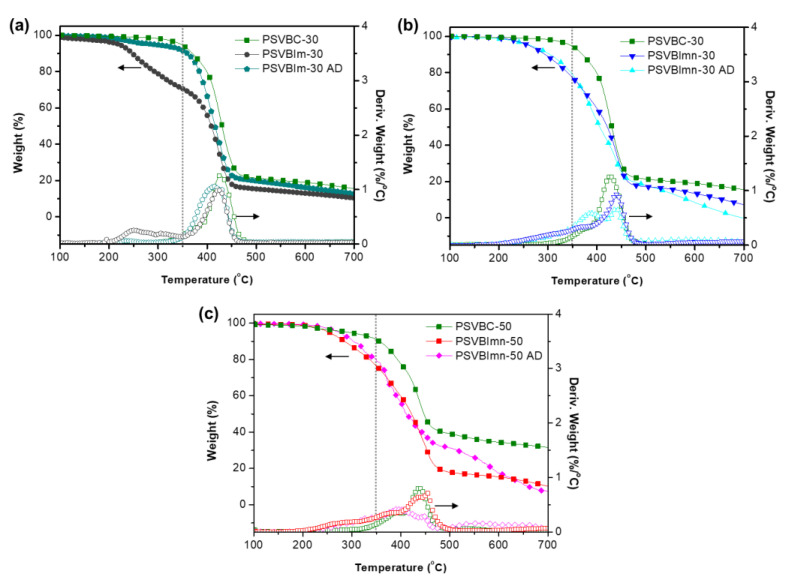
TGA thermograms of the (**a**) PSVBIm-30, (**b**)PSVBImn-30, and (**c**) PSVBImn-50 membranes before and after the alkaline treatment in 1 M KOH at 60 °C for 144 h. (AD: alkaline degradation).

TGA was employed to detect the change in compositions due to alkaline degradations of the AEMs by comparing their thermograms before and after the alkaline treatment. Figure 5 presents the TGA curves recorded from 100 to 700 °C. The first weight loss in the temperature range lower than 350 °C was attributed to the thermal degradation of either imidazolium or imidazolinium heterocycles. The decompositions of polystyrene copolymers (PSVBC-30 and PSVBC-50) contributed to the weight loss at temperatures approximately higher than 350 °C.

Figure 5a compares the thermograms of PSVBC-30, PSVBIm-30, and PSVBIm-30 treated with alkaline degradation (abbreviated as PSVBIm-30 AD). It revealed that the first weight loss corresponding to the thermal decomposition of imidazolium reduced significantly for PSVBIm-30 AD. This result accords with the finding of the hydroxide conductivity decay during the alkaline treatment for the PSVBIm-30 membrane. In contrast, we observed no significant difference between the thermograms before and after alkaline treatment for whether PSVBImn-30 or PSVBImn-50, especially at temperatures below 350 °C, as shown in Figure 5b,c. This observation indicated that the alkaline degradations of PSVBImn-30 and PSVBImn-50 were limited, which supports the minor change in hydroxide conductivity for these two AEMs under strongly basic conditions.

It is known that a crosslinked structure is beneficial for improving the chemical, thermal and dimensional stabilities of a polymer membrane [3,36]. Aside from that, several studies suggested that the crosslinked structure is capable of further enhancing the alkaline stability of AEMs [37,38,39]. In the present work, the crosslinked networks bridged with the unsaturated oleyl chains may protect the membranes from the hydroxide ion attacks, resulting in good alkaline stability. Conversely, previous studies suggested that the C2-substitution of imidazole derivatives can increase the steric interference on C2 position and enhance the resistance to the attack of hydroxide ions that undergo via the S_N_2 reaction [40,41]. In our case, HEOImn consists of a C2-substituted unsaturated oleyl chain, but 1-methylimidazole lacks a C2-substituted group. This reason can explain why the imidazolium cations grafting onto PSVBIm-30 were prone to degrade more easily in alkaline conditions than the imidazolinium cations attached to PSVBImn-30 and PSVBImn-50.

**Figure 6:**
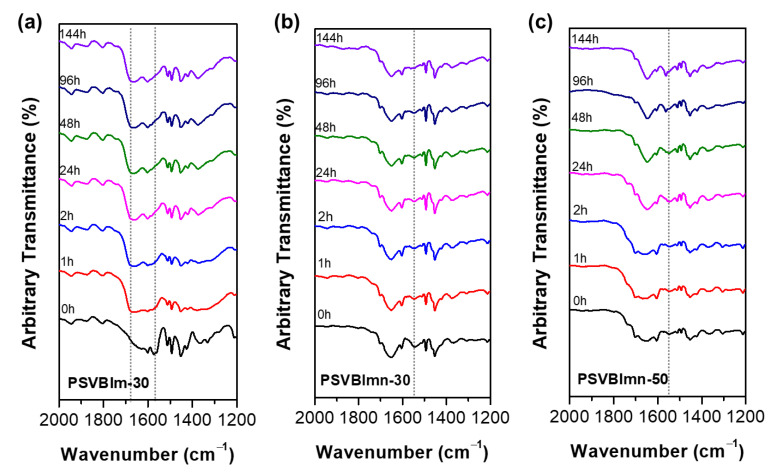
Evolution of FTIR spectra of the (**a**) PSVBIm-30, (**b**) PSVBImn-30, and (**c**) PSVBImn-50 membranes during the alkaline treatment in 1 M KOH at 60 °C for 144 h.

Furthermore, we used FTIR analysis to identify any changes in chemical structure caused by alkaline degradations of AEMs [42]. Figure 6a shows the FTIR spectra of PSVBIm-30 during the 144 h alkaline treatment, which presented that the characteristic peak of imidazolium at 1572 cm^−2^ lessened its absorption intensity considerably. In the meantime, an additional peak at around 1690 cm^−2^ was found to rise gradually, which is associated with the presence of tertiary amide groups belonging to the ring-opening product of imidazolium. This result confirmed that the 1-methylimidazolium cations of PSVBIm-30 underwent a ring-opening decomposition during the alkaline treatment.

**Scheme 4:**
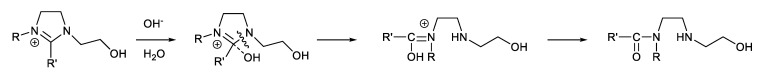
Proposed procedures for the ring-opening of imidazolinium cations in the presence of hydroxide ions.

Imidazoline compounds can be hydrolyzed in a basic aqueous solution via a ring-opening reaction to form amine-amide compounds [24]. If the alkaline degradation of imidazolinium cations causes ring-opening in the same way, the product will be compounds containing secondary amine and tertiary amide groups [43], as depicted in Scheme 4. The proposed ring-opening mechanism of imidazolinium during the alkaline degradation is similar to the ring-opening mechanism of imidazolium, as suggested in the literature [44,45].

However, no big changes in the evolutionary FTIR spectra within 48 h for PSVBImn-30 and PSVBImn-50 were observed, as shown in Figure 6b,c. In addition, the possible absorption peak in the range from 1630 cm^−1^ to 1670 cm^−1^ corresponding to the C=O stretching vibrations of tertiary amide was not found. The changes in hydroxide conductivities, thermograms, and FTIR spectra for both the PSVBImn-30 and PSVBImn-50 membranes were insignificant, suggesting that imidazolinium cations are alkaline stable.

### 3.5. Fuel Cell Performance

**Figure 7:**
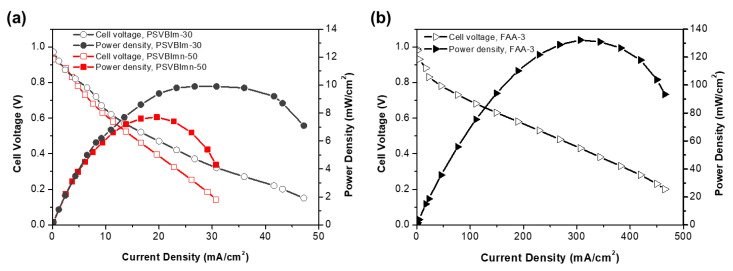
Polarization curves of the MEAs based on (**a**) the PSVBIm-30 and PSVBImn-50 membranes as well as (**b**) the commercially available AEM FAA-3.

The fuel cell test was conducted to evaluate the application potential of the AEM for AEMFCs. The polarization curves for the MEAs based on the PSVBIm-30 and PSVBImn-50 membranes were recorded at 60 °C without back pressure, as shown in Figure 7a. The open circuit voltages of fuel cell (approximately 0.97 V) were close to the theoretical OCV value at 60 °C (1.20 V) [46], indicating that the fuel crossover problem for this MEA was limited. However, the peak power densities for PSVBIm-30 and PSVBImn-50 were measured to be only 9.9 and 7.8 mW/cm^2^, which is much lower than the values reported recently for other AEMs containing imidazolium cations [47,48,49]. To confirm if the poor fuel cell performance was obtained under appropriate conditions or not during the MEA fabrication and the AEMFC operation, we carried out an additional fuel cell test for the MEA based on the commercially available FAA-3 (Fumatech) AEM that was fabricated using identical catalyst ink and following the same procedures. The chemical structure of FAA-3 consists of a poly(phenylene oxide) backbone with quaternary ammonium functionality [50]. As presented in Figure 7b, the peak power density for the FAA-3 membrane at 60 °C reached 132 mW/cm^2^, which is comparable to the reported values [51]. In addition, its polarization curves exhibited a much lower slope in the linear portion corresponding to the ohmic resistance compared with the polarization curves for PSVBIm-30 and PSVBImn-50. The ohmic resistance is directly dependent on the hydroxide conductivity for an AEM. The hydroxide conductivity at 60 °C of FAA-3 was measured to be 23.7 mS/cm, which was higher than that of PSVBImn-50 (5.5 mS/cm). The relatively low hydroxide conductivity of the PSVBImn-50 membrane would be the major reason for the insufficient power density of its AEMFC. However, the low power density for PSVBIm-30 was possibly due to the insufficient alkaline stability in a basic media environment at relatively high temperatures.

## 4. Conclusions

In this work, imidazole quaternized and imidazoline quaternized polystyrene copolymers PSVBIm-30, PSVBImn-30, and PSVBImn-50 were successfully synthesized, and their chemical structures were confirmed by the joint results of FTIR and ^1^H-NMR. The PSVBIm-30 membrane exhibited higher water uptake, swelling ratio, and hydroxide conductivity than the PSVBImn-30 and PSVBImn-50 membrane due to its higher IEC. The hydroxide conductivities of the PSVBImn-30 and PSVBImn-50 membranes respectively reached 5.81 and 3.13 mS/cm, suggesting that imidazolinium cations are feasible to be a new type of cation for AEMs. Moreover, the imidazolinium-based AEMs were found alkaline stable, evidenced by the insignificant changes in their hydroxide conductivities, TGA thermograms and FTIR spectra after alkaline treatment. Compared with PSVBImn-30 and PSVBImn-50, the imidazolium-based AEM PSVBIm-30 prepared in the present work exhibited relatively poor alkaline stability. N-b-hydroxyethyl oleyl imidazolinium cations are supposed to be more alkaline stable than 1-methylimidazolium cations. Aside from the cation effect, the crosslinked networks and the steric interference on C2 position of imidazolinium would enhance the imidazolinium-based AEM’s alkaline stability. However, the insufficient hydroxide conductivity of the PSVBImn-50 membrane would bring a negative effect on its AEMFC performance. Its highest power density recorded at 60 °C was only 7.8 mW/cm^2^.

## Figures and Tables

**Table 1:** Composition, molecular weight, and polydispersity of PSVBC-30 and PSVBC-50.

Copolymer	St/VBC Molar Ratio ^1^	Mn ^2^	Mw ^2^	PDI ^3^
PSVBC-30	66.6/33.4	3.67 × 10^4^	5.40 × 10^4^	1.47
PSVBC-50	50.7/49.3	3.77 × 10^4^	7.31 × 10^4^	1.94

^1^ calculated from the peak integration area of the NMR spectra. ^2^ the number-average molecular weight (Mn) and the weight-average molecular weight (Mw) of a polymer are measured by GPC analysis. ^3^ polydispersity (PDI) is defined as the ratio of Mn to Mw.

**Table 2:** Ion exchange capacity, water uptake, swelling ratio and gel fraction of the PSVBIm-30, PSVBImn-30, and PSVBImn-50 membranes in OH^‒^ form.

AEM	Theoretical IEC ^1^ (mmol/g)	Experimental IEC ^2^ (mmol/g)	WU ^3^ (%)	SR ^3^ (%)	Gel Fraction (%)
PSVBIm-30	1.96	1.88	124.0 ± 2.2	37.5 ± 3.2	0
PSVBImn-30	1.32	1.11	84.6 ± 6.9	4.0 ± 0.8	30
PSVBImn-50	1.63	1.63	210.7 ± 3.0	13.9 ± 2.1	55

^1^ calculated according to the St/VBC molar ratio of the PSVBC copolymer. ^2^ determined by the back-titration method. ^3^ measured at 60 °C.

**Table 3:** A comparison of the hydroxide conductivities of the AEMs based on poly(St-co-VBC) containing different cationic groups.

AEM	St/VBC Molar Ratio (mol%)	Experimental IEC (meq g^−1^)	Cationic Group	Measured Temperature (°C)	Hydroxide Conductivity (mS cm^−1^)	Ref.
QMSV-0.33	75/25	2.14	quaternary ammonium	25	6.8	[27]
Poly(St-co-VBMI)	71.9/28.1	1.96	imidazolium	30	7.8	[7]
PSVBIm-30	66.3/33.4	1.88	imidazolium	30	15.8	This work
PSVBImn-30	66.3/33.4	1.11	imidazolinium	30	0.8	This work
PSVBImn-50	50.7/49.3	1.63	imidazolinium	30	3.9	This work
Copolymer A	80.9/19.1	1.45	imidazolium	30	12.4	[2]
Membrane-1 (TMA)	75/25	1.07	quaternary ammonium	-	1.9	[35]
